# Perceptions of Proactive Palliative Care Integration Among Pediatric Hematopoietic Cell Transplant Providers: A Pilot Study

**DOI:** 10.3390/children13070854

**Published:** 2026-06-26

**Authors:** Sydney Ariagno, Vida Alami, Dexiang Gao, Kristen Eisenman, Mary Benson, Vanessa A. Fabrizio, Adam B. Hill, Jenna Demedis

**Affiliations:** 1 Department of Pediatrics, University of Colorado School of Medicine, Aurora, CO 80045, USA; 2 Cancer Center Biostatistics and Bioinformatics Shared Resource, University of Colorado School of Medicine, Aurora, CO 80045, USA; 3 Pediatric Palliative Medicine, Children’s Hospital Colorado, Aurora, CO 80045, USA; 4 Center for Cancer and Blood Disorders, Children’s Hospital Colorado, Aurora, CO 80045, USA

**Keywords:** pediatric palliative care, hematopoietic cell transplant, oncology, care delivery, palliative care implementation, perceptions of palliative care

## Abstract

Background: Pediatric hematopoietic cell transplantation (HCT) conveys significant risk of mortality, morbidity, impaired quality of life, and multifactorial distress. One potential strategy for improving experience and relieving suffering is proactive specialty palliative care (SPC) utilization. However, SPC is not routinely incorporated into pediatric HCT. One barrier to SPC integration is unknown pediatric HCT provider perceptions of SPC services, particularly among providers with lived experience working within a collaborative HCT-SPC partnership. Objective: This single-institution pilot study aimed to (1) describe an approach to standardized, proactive pediatric HCT-SPC clinical partnership, and (2) quantify acceptability, appropriateness, and satisfaction regarding the program among pediatric HCT providers. Methods: Survey methods were used to assess attitudes among HCT providers who had worked with the SPC clinical partnership for at least three months. Core survey metrics were the validated Acceptability of Intervention Measure and Intervention Appropriateness Measure. Additional survey items were adapted from the Perceptions of Palliative Care Instrument. Results: Respondents reported high mean scores for acceptability (4.96) and appropriateness (4.93) on a 5-point scale. Overall satisfaction with SPC integration averaged 8.72 (SD 1.13) on a 10-point scale. Satisfaction scores for each individual service provided by SPC were similarly high. No significant differences in responses were found based on provider type, prior SPC training, or years in practice. Conclusions: In this single-institution pilot study, pediatric HCT providers with lived experience working in an environment with standardized SPC collaboration view SPC as highly acceptable, appropriate, and beneficial for their patients, supporting the feasibility and value of proactive SPC integration in pediatric HCT care.

## 1. Introduction

Hematopoietic cell transplantation (HCT) is a critical component of curative treatment for pediatric malignancies, disorders of hematopoiesis, immunodeficiencies, and metabolic diseases. Despite advancements in safety and efficacy, pediatric HCT continues to confer substantial risk. All-cause mortality in the first five years following HCT is approximately 10–20% [1,2,3,4]. Numerous complications, such as veno-occlusive disease, graft-versus-host disease, or bronchiolitis obliterans, threaten survivors’ quality of life and can lead to prolonged hospitalizations or intensive care unit admissions [5,6,7,8]. Pediatric patients and their families also experience significant distress related to therapy itself. Physical symptoms (including pain, fatigue, nausea, dyspnea, anorexia, and insomnia) are common and correlate with decreased quality of life [9]. Emotional distress, neurocognitive changes, and poor mental health are well documented amongst both patients and caregivers during treatment [10,11,12,13,14,15]. Lastly, suffering secondary to financial, spiritual, practical, and familial concerns is pervasive as well [11,16,17].

One potential strategy for improving quality of life and relieving distress for pediatric HCT recipients is proactive integration of palliative care. Specialty palliative care (SPC) is a multidisciplinary model of team-based care focused on improving quality of life for patients with potentially life-limiting illnesses and/or embarking on medical therapies with high risk of morbidity and mortality [18]. Despite American Society for Transplantation and Cellular Therapy (ASTCT) recommendations to involve SPC for all children undergoing HCT for malignant indications, integration of SPC in pediatric HCT care is not widely adopted [19]. Investigation regarding its potential benefits is in early stages, focusing primarily on SPC impact on end-of-life care for pediatric HCT patients [20]. Lack of abundant data regarding benefits in pediatrics, HCT provider attitudes towards SPC, and poor understanding of its role may be barriers to adherence to this ASTCT guideline.

National, multicenter survey data exists detailing general perceptions of SPC among HCT providers [21,22]. Adult and pediatric HCT providers alike agree that their patients have unmet needs that SPC can address. However, they also worry that SPC may not be well received by patients and that SPC providers may not have sufficient HCT knowledge to care for this unique population. A drawback of these national surveys is that programmatic integration between SPC and HCT is highly varied between institutions. These data represent opinions of HCT providers with diverse lived experiences working with SPC, ranging from intensive partnerships to little or no overlap in care. No literature currently exists detailing pediatric HCT provider perceptions following implementation of a rigorous, active clinical partnership between the two fields.

Here, we describe one pilot approach to standardized, proactive SPC engagement within pediatric HCT care at a single, large-volume institution. Following implementation of this program at the study institution, we quantified implementation metrics (acceptability and appropriateness) as perceived by the pediatric HCT providers working with this partnership program. We measured overall satisfaction with SPC integration, as well as elucidated specific perceptions of individual SPC services. Lastly, we identified HCT provider beliefs regarding attitudes of patients and their families towards SPC.

## 2. Methods

### 2.1. Description of the Pilot SPC Intervention

In accordance with ASTCT societal recommendations, our institution developed a standardized, integrated SPC intervention, which is provided for all pediatric allogeneic HCT recipients as part of routine clinical care starting in January 2024. The study institution is a large-volume academic children’s hospital in the United States Mountain Region, with access available to all pediatric subspecialties and serving as the primary pediatric HCT provider for seven states. The SPC team at the study institution (during the period the study was conducted) consists of five physicians, three nurse practitioners, one social worker, and two specialized registered nurses, each with advanced training or specialty certification in SPC. While all patients considering allogeneic HCT are referred to receive SPC, the frequency and cadence of SPC visits for HCT patients follows a non-prescribed pattern that allows for individualization at the discretion of SPC providers and family. Prior to implementation of this pilot program, the practice of automated pre-HCT referral did not occur at the study institution and referral to SPC was on a case-by-case basis.

**Figure 1:**
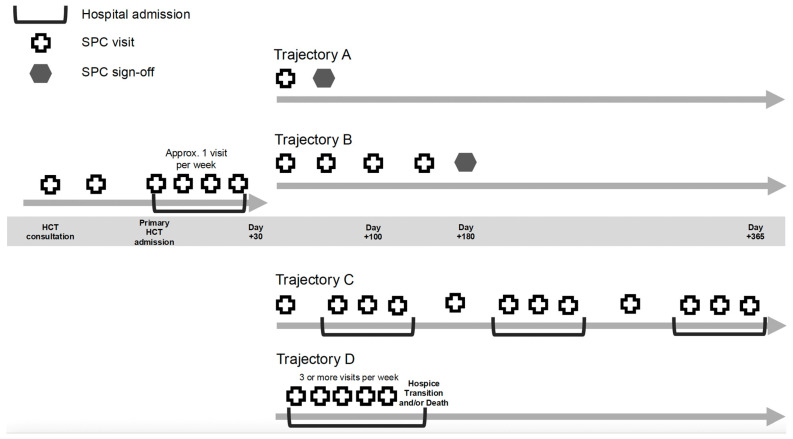
Timeline of typical SPC consultation and follow-up visitation for HCT patients, based on clinical trajectory. Figure Key: Trajectory A: Good prognosis, no complications that threaten QOL or may be life-limiting, physical symptoms resolved. SPC team may even sign off at time of primary hospital discharge. Trajectory B: Likely good prognosis, stable out of the hospital, but with ongoing complications that threaten QOL or may be life-limiting, physical symptoms ongoing. Cadence and frequency of these visits determined with familial preference incorporated. Trajectory C: Worrisome prognosis, frequent readmissions, severe complications that threaten QOL and/or are life-limiting, active medical decision-making, notable physical symptoms. Trajectory D: Poor prognosis, elevated risk of acute mortality, ICU admissions, poor QOL, active medical decision-making, notable physical symptoms.

The timeline for SPC integration and follow-up visits is depicted in Figure 1. When a pediatric patient is referred for allogeneic HCT at the study institution, the assigned primary HCT provider requests SPC consultation. An initial pre-HCT SPC consultation occurs via telemedicine at minimum 1–2 weeks prior to admission for HCT. The focus of this initial consultation is aimed at establishing a therapeutic relationship with the family, identifying hopes and worries for HCT, detailing current symptom burden, considering proactive strategies to manage anticipated symptoms, discerning value-based goals of care, introducing advance care planning, identifying surrogate decision-makers, and providing education about the role of the SPC team as an additional layer of support for the family during the challenges of HCT. When clinically indicated, the SPC team may conduct subsequent outpatient visits prior to HCT admission, to assist with decision-making regarding the choice to pursue HCT or other active concerns. Upon admission for HCT, the SPC team visits the patient within 1–2 days of admission and then approximately once every week, with the ability to increase or decrease frequency depending on the clinical scenario. Following discharge from primary HCT admission, patients tend to follow one of four pathways. For patients discharged with no comorbidities and excellent functional status, SPC will sign off, with the reminder that they can be re-engaged at any time should the clinical situation change (Trajectory A). For patients who are stable outside of the hospital but continue to experience comorbidities that are anticipated to represent an ongoing threat to quality of life, SPC will continue to see the patient via telemedicine outside of the hospital for as long as clinically indicated (Trajectory B). For patients that experience early or frequent readmission to the hospital due to complications, SPC will be re-consulted with each hospital stay and will continue to visit the patient at the previously described cadence. SPC will also provide telehealth outpatient visits for these patients if/when they are discharged from the hospital (Trajectory C). Lastly, if at any point the patient develops a complication that acutely increases risk of transplant-related mortality, SPC involvement increases to multiple visits per week and will continue to provide frequent care through end of life (Trajectory D).

At the time this study was conducted, the services provided by the SPC team at the study institution included advance care planning, elucidation of goals of care, assistance with complex medical decision-making, provision of psychosocial support, and expertise in end-of-life care. SPC services were available weekdays during business hours. Comprehensive physical symptom management was not included in their scope of practice and was managed by the HCT team or the anesthesia acute pain service; however, SPC providers did occasionally provide recommendations related to physical symptom management for complex patients.

To provide information about program delivery, a random sampling of patients who underwent HCT in the first year after pilot program initiation were identified using random number generation and specific SPC delivery metrics were evaluated using descriptive statistics.

### 2.2. Survey Methods

Providers (physicians, nurse practitioners, physician assistants) who regularly provide care to patients undergoing allogeneic HCT and have participated in combined care with the SPC team for at least three months were eligible to participate in the study. REDCap surveys were distributed via email to all eligible providers cross-sectionally, approximately one year following the initiation of the HCT-SPC clinical partnership. Participants had a total of four weeks to complete their responses. Survey responses were deidentified. Participants were compensated with a $10 gift card following survey completion.

The survey in its entirety is available in Supplemental File S1. Core metrics that represent the overarching impression of the SPC clinical partnership are the Acceptability of Intervention Measure (AIM) and Intervention Appropriateness Measure (IAM), which are validated implementation outcome measures [23]. The AIM and IAM tools are scored on a scale from 1 to 5, with scores of 4 to 5 indicating high levels of acceptability and appropriateness. Additional survey items were developed using the Perceptions of Palliative Care Instrument, as well as other published survey protocols exploring HCT and oncology providers’ attitudes towards SPC services [22,24,25]. Choices for survey item inclusion from the adapted existing questionnaires were made by expert review with opinions from both HCT and SPC teams represented. Surveys were piloted with three respondents prior to wide dissemination to ensure clarity and understanding. Survey questions are all quantitative with either a numerical rating system or Likert-style response options.

This study, which is considered no more than minimal risk to participants, was reviewed and approved by the Colorado Multiple Institutional Review Board. Informed consent was obtained by provision of an information sheet at the beginning of the written survey.

### 2.3. Statistical Analysis

Descriptive statistics, including frequencies and percentages for categorical variables and means and standard deviations for continuous variables, were utilized. Participants were stratified into groups based on certain provider characteristics to assess whether survey responses were associated with those characteristics. The groups were determined a priori, and the characteristics included provider type (physician vs. advanced practice provider), prior palliative care training (some prior training or work vs. none), and years in practice (0–5 years vs. greater than 5 years). Likert-style survey items were treated as categorical variables and evaluated for association with provider groups using Fisher’s exact test. A *p*-value of <0.05 was considered statistically significant.

## 3. Results

### 3.1. Description of HCT-SPC Program Delivery

In a sampling of twenty random patients who received allogeneic HCTs at our institution in the last year, there was a median of 8.5 (range 5–22) separate encounters with SPC between initial HCT referral consult and time of either SPC sign-off or patient death. Median encounter length was 50 (range 25–120) minutes. Two of twenty patients (10%) had SPC involved in their care prior to HCT referral. Families did have the option to decline initial SPC consultation or ongoing SPC involvement; however, no families were noted to have declined SPC services at time of data collection.

### 3.2. Participant Demographics

Eighteen providers were eligible to participate, and 100% completed the survey. Full demographic information for the participants is available in Table 1. Median age of the cohort was 37, ranging from 27 to 59. Half of the participants were attending physicians, with the other half divided between nurse practitioners, physician assistants, and fellow physicians. Median years since completion of clinical training was 5 years (range 0–25). Seven participants (39%) reported no prior work or training with SPC.

### 3.3. Acceptability and Appropriateness Measures

Across all respondents, the mean composite acceptability (AIM) rating was 4.96 (median 5, range 4.5–5), and the mean composite appropriateness (IAM) rating was 4.93 (median 5, range 4–5). Ratings across individual questions for both metrics were similarly high (Table 2). No scores of 1–3 (which would indicate a poor perception of acceptability or appropriateness) were indicated by any participant. All respondents agreed that early SPC involvement seemed ideal for pediatric HCT patients, indicating that the best time for SPC consultation is either at the time that HCT is being discussed as a possible treatment option (72%) or at the time of initial HCT consultation (28%). No participants selected a later time point as the ideal time for SPC involvement. There was no significant association found between AIM or IAM scores and provider type, prior palliative care training, or years in practice.

### 3.4. Satisfaction with SPC Services

**Figure 2:**
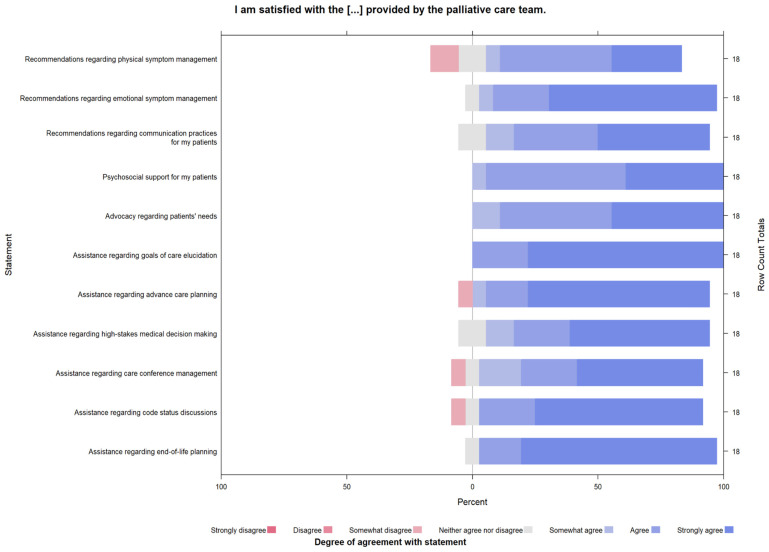
Satisfaction with various domains of SPC services.

The mean rating of overall satisfaction with SPC integration (on a scale from 1 to 10; higher scores correspond to greater satisfaction) was 8.72 (SD 1.13, range 7–10). Participants were asked to indicate their degree of satisfaction with individual services provided by the SPC team on a scale of 1–7 (higher scores correspond to greater satisfaction). Almost all domains assessed demonstrated a mean score of 6 or greater, with the exception of physical symptom management (mean 5.7, SD 1.3) (Figure 2). The domains garnering the highest degree of satisfaction among participants were goals of care elucidation (mean 6.8, SD 0.4), end-of-life planning (mean 6.7, SD 0.8), and advance care planning and emotional symptom management (both mean 6.5, SD 1.0 and 0.9, respectively). No significant association between any satisfaction scores and provider type, prior palliative care training, or years in practice were detected.

### 3.5. Beliefs Regarding Patient/Family Perceptions of SPC

**Figure 3:**
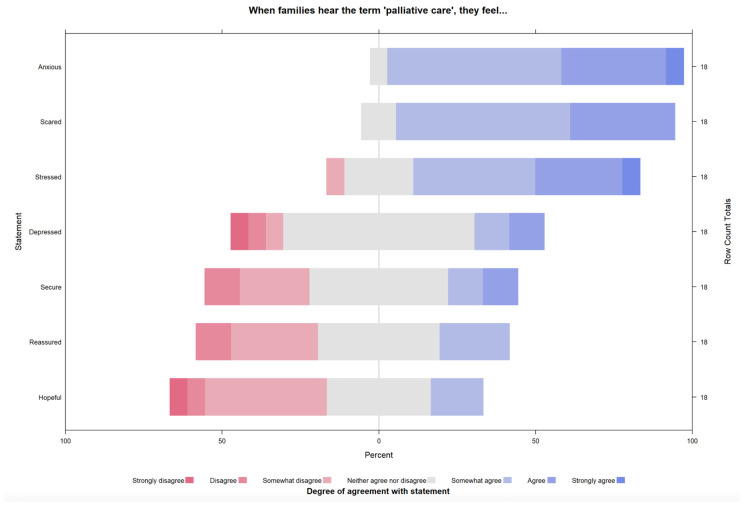
Provider perceptions of how families feel when hearing the term “palliative care”.

**Figure 4:**
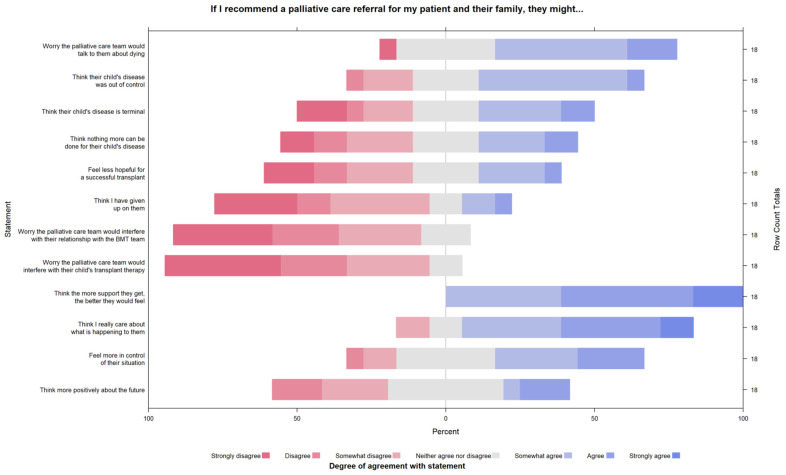
Additional provider perceptions of familial beliefs about palliative care.

Participants were asked to indicate their degree of agreement related to how introduction to SPC may make a patient and/or family feel. Overall, respondents somewhat agreed that introduction to SPC would make patients/families feel anxious, scared, or stressed; they somewhat disagreed that introduction to SPC would make patients/families feel secure, reassured, or hopeful (Figure 3). Participant responses to additional questions related to patient/family perceptions of SPC are available in Figure 4. Notable findings include agreement that upon introduction of SPC, families would think the more support they get, the better they will feel, as well as that families would think their HCT provider really cares about what happens to them. Additionally, respondents did not believe that families would worry that SPC involvement would interfere with their relationship with the HCT team or with their HCT therapy. Respondents indicated mixed perceptions regarding if SPC introduction would cause families to feel less hopeful for a successful HCT. There was a slightly statistically significant association between years in practice and the perception that SPC involvement makes families feel reassured (*p* = 0.046). Those in practice longer than 5 years tended towards mild disagreement with this statement, whereas those in practice 0–5 years tended to respond neutrally to this statement. Otherwise, no significant associations in any of these domains were detected when comparing by provider type, prior palliative care training, or years in practice.

## 4. Discussion

Early, standardized SPC involvement has been explored in multiple general pediatric oncology settings and has been demonstrated to be a well-accepted intervention that reduces distress, improves quality of life, and aids families in complex medical decision-making [26,27,28]. SPC integration among adult HCT patients has been documented as similarly beneficial. El-Jawahri et al. demonstrated via a randomized controlled trial that adult HCT patients who had upfront SPC involvement, as compared to those receiving routine care, had higher quality-of-life scores at two weeks and six months following HCT [29,30]. In the limited amount of pediatric HCT data available, SPC involvement is associated with de-intensified end-of-life care, with decreased utilization of intensive care services and decreased code-level interventions [20]. These findings serve as the foundation behind efforts and societal recommendations to routinely integrate SPC into pediatric HCT care. Widespread implementation, however, is currently lacking.

Here we describe one model of standardized SPC integration for pediatric HCT recipients. This model offers multiple potential benefits. First, the ability to introduce SPC as a resource recommended for all families undergoing HCT, regardless of illness status or indication for HCT, allows for normalization of SPC utilization and may increase acceptance of or decrease negative emotion surrounding SPC for families. Second, using standardized referral criteria for a predetermined patient population has been previously cited as a potential way to improve HCT/SPC collaboration [31]. Third, flexibility of visit cadence depending on clinical scenario allows the SPC team to tailor their services in an individualized fashion to meet unique patient needs.

One potential barrier to proactive SPC utilization is pediatric HCT provider perceptions regarding the utility of SPC services and beliefs regarding patient and family acceptance of such an intervention [21,22,31,32]. Here we show that among pediatric HCT providers working in an environment with standardized SPC collaboration, provider perceptions of SPC are universally positive. Validated metrics of acceptability and appropriateness for proactive SPC integration for all allogeneic HCT recipients demonstrated uniformly high scores amongst our cohort, without outliers. Overall satisfaction ratings, as well as satisfaction with individual services provided by SPC, were also unvaryingly positive. These findings demonstrate that HCT providers who have lived experience with an institutional SPC collaboration see value in SPC utilization and feel that it has been impactful for their patient population.

Notably, we describe these positive perceptions of SPC coexistent with HCT provider concerns about how families feel about SPC. Participants noted worry that SPC introduction may cause negative emotions or opinions in pediatric HCT families. These findings are similar to those described in prior national surveys of HCT providers [21,22]. However, limited existing literature suggests that these HCT provider concerns may be discrepant with patient and family actual beliefs. Feasibility studies examining SPC referral in the HCT population demonstrate high levels of acceptance of SPC involvement, interest in early SPC consultation, and desire for greater focus to be placed on quality of life during the HCT process [33,34,35]. Study of pediatric HCT patients’ and families’ perceptions of integrated SPC within our clinical program is ongoing.

A prior national survey of HCT providers identified the service name of “palliative care” as a barrier to referral and preferred the term “supportive care”. In adult HCT and oncology contexts, change of the service name to “supportive care” is associated with earlier and increased referrals to SPC [36,37]. Naming conventions for SPC teams is a source of debate in pediatrics, though it has been discussed that efforts should center on increasing education surrounding SPC, rather than changing its title [21]. Our findings suggest that HCT/SPC integration programs can be well received by providers and have high rates of family participation while continuing to utilize the traditional name of “palliative care”.

This study carries the limitation of a small sample size at a single institution. Due to the varied nature of SPC models, services, and resources across institutions, the generalizability of our findings is reduced. The completion rate of 100% does improve the validity of our findings (though does not completely mitigate limited generalizability) as the perceptions of our entire HCT provider team are represented in these findings, and we have avoided voluntary response bias. We also acknowledge the likelihood of social desirability bias potentially inflating results. Efforts were taken to minimize this influence, including use of validated survey tools, complete anonymization of responses, and completion of the survey by electronic form, rather than written or in-person administration. Additionally, at the time of study completion, institutional staffing limitations constrained the SPC team’s ability to provide direct symptom management. Physical symptom management is a common service provided by SPC teams, and while this was not directly explored, we hypothesize that lack of this comprehensive resource may be the driving cause for “physical symptom management” scoring relatively low in the satisfaction with SPC services analysis (Figure 2). This barrier to comprehensive SPC provision at our institution has been mitigated by the hiring of two additional physicians since the time this study was conducted. Lastly, small sample size may have limited our ability to detect significant differences between demographic comparison groups due to low power; subgroup comparisons should be interpreted cautiously and warrant further study.

Overall, we demonstrate that proactive SPC integration for pediatric HCT patients is perceived as a highly acceptable, appropriate, and satisfactory intervention among HCT providers in this single-institution pilot study. To the best of our knowledge, this is the first study detailing perceptions of SPC exclusively among pediatric HCT providers who have lived experience working in a care environment with early and ongoing SPC integration for their patients. As demonstrated in previous survey-based studies, our participants did express worry regarding how SPC is received by patients and families. Future research is needed to examine what families’ reactions to SPC are upon initial introduction, as well as detail the impact of proactive SPC integration on key domains such as quality of life, symptom management, distress experience, and decision-making during HCT.

## Figures and Tables

**Table 1:** Participant demographics.

Characteristic	N = 18
**Age**	
Median (range)	37 (27, 59)
Mean (SD)	38 (8)
**Gender**	
Woman	10 (56%)
Man	7 (39%)
Nonbinary	1 (5.6%)
**Race**	
White	13 (72%)
Asian, White	3 (17%)
Asian	2 (11%)
**Ethnicity**	
Not Hispanic or Latino	18 (100%)
**Type of provider**	
Attending physician	9 (50%)
Nurse practitioner	4 (22%)
Physician assistant	4 (22%)
Fellow physician	1 (5.6%)
**Portion of FTE dedicated to clinical responsibilities**	
Median (range)	80 (20, 100)
Mean (SD)	72 (30)
**Years in practice since training completion**	
Median (range)	5.0 (0.0, 25.0)
Mean (SD)	6.1 (6.1)
**Prior formal training or work completed in palliative care**	
Dedicated rotation during residency/fellowship/advance practice training	7 (39%)
No prior training or work	7 (39%)
Attended CME courses and/or educational lectures	2 (11%)
Dedicated rotation during residency/fellowship/advance practice training	2 (11%)
Attended CME courses and/or educational lectures	
n (%)	

**Table 2:** Mean AIM and IAM Scores.

AIM Scores	
Please Indicate the Degree to Which You Agree with the Following Statements	N = 18
Standardized palliative care integration for allogeneic BMT patients meets my approval	4.9 (0.3)
Standardized palliative care integration for allogeneic BMT patients is appealing to me	4.9 (0.2)
I like standardized palliative care integration for allogeneic BMT patients	5.0 (0.0)
I welcome standardized palliative care integration for allogeneic BMT patients	5.0 (0.0)
Mean score for the acceptability section: 4.96	
Mean (SD)	
**IAM Scores**	
**Please Indicate the Degree to Which You Agree with the Following Statements**	N = 18
Standardized palliative care integration for allogeneic BMT patients seems fitting	4.9 (0.2)
Standardized palliative care integration for allogeneic BMT patients seems suitable	4.9 (0.2)
Standardized palliative care integration for allogeneic BMT patients seems applicable	4.9 (0.2)
Standardized palliative care integration for allogeneic BMT patients seems like a good match	4.9 (0.3)
Mean score for the appropriateness section: 4.93	
Mean (SD)	

## Data Availability

The original contributions presented in this study are included in the Supplementary Material. Further inquiries can be directed to the corresponding author.

## Supplementary Materials

The following supporting information can be downloaded at: https://www.mdpi.com/article/10.3390/children13070854/s1, Supplementary File S1: Pediatric HCT Provider Attitudes Towards Early Palliative Care Involvement Complete Survey; Supplementary File S2: Raw Survey Data Results.
