# Supplementary material for: Perceptions of Proactive Palliative Care Integration Among Pediatric Hematopoietic Cell Transplant Providers: A Pilot Study
Source: Children (Basel). 2026 Jun 26;13(7):854. doi: 10.3390/children13070854 (PMC13406733; doi:10.3390/children13070854)
Supplement: Supplementary file 1 [file children-13-00854-s001.zip › children-4360387-File S1.pdf]

Provider Attitudes Survey
COMIRB # 24-1913
PI: Sydney Ariagno, MD
Version: 1.0
Version Date: 10-29-2024

Supplementary File S1. **Pediatric HCT Provider Attitudes Towards Early Palliative Care Involvement**

Demographics

Age (years): ______

Gender:
- Male
- Female
- Nonbinary
- Other: __________
- Prefer not to say

Ethnicity
- Hispanic or Latino
- Not Hispanic or Latino
- Unknown or prefer not to say

Race (select all that apply):
- American Indian or Alaska Native
- Asian
- Black or African American
- Hispanic or Latino
- Native Hawaiian or Other Pacific Islander
- White
- Unknown or prefer not to say

Select the option that best describes what type of provider you are:
- o Attending physician
- o Fellow physician
- o Nurse practitioner
- o Physician assistant
- o Prefer not to answer

What portion of your FTE dedicated to clinical responsibilities? ___________

How many years have been in practice since completion of your training? ___________

Select any of the following options that best describe any prior formal training or work you completed in palliative care (check all that apply):
- o No prior training or work

- Dedicated rotation during residency/fellowship/advance practice training
- Attended CME courses and/or educational lectures
- Completed specialized palliative care fellowship
- Worked in specialized palliative care or hospice setting for >6 months
- Prefer not to answer

Acceptability of Intervention Measure

Core measure

Please indicate the degree to which you agree with the following statements (response options include: completely disagree, disagree, neither agree nor disagree, agree, and strongly agree)

Standardized palliative care integration for allogeneic BMT patients meets my approval
Standardized palliative care integration for allogeneic BMT patients is appealing to me
I like standardized palliative care integration for allogeneic BMT patients
I welcome standardized palliative care integration for allogeneic BMT patients

Intervention Appropriateness Measure

Core measure

Please indicate the degree to which you agree with the following statements (response options include: completely disagree, disagree, neither agree nor disagree, agree, and strongly agree)

Standardized palliative care integration for allogeneic BMT patients seems fitting
Standardized palliative care integration for allogeneic BMT patients seems suitable
Standardized palliative care integration for allogeneic BMT patients seems applicable
Standardized palliative care integration for allogeneic BMT patients seems like a good match

Satisfaction with Specific Palliative Care Services

Please rate your overall impression of the quality of services provided by the palliative care team (0 = lowest quality, 10 = highest quality)

Please indicate the degree to which you agree with the following statements (response options include: strongly disagree, disagree, somewhat disagree, neither agree nor disagree, somewhat agree, agree, and strongly agree)

"I am satisfied with the recommendations provided by the palliative care team regarding physical symptom management."
"I am satisfied with the recommendations provided by the palliative care team regarding emotional symptom management."
"I am satisfied with the recommendations provided by the palliative care team regarding communication practices for my patients."
"I am satisfied with the psychosocial support the palliative care team provides my patients."
"I am satisfied with the advocacy provided by the palliative care team regarding patients' needs."
"I am satisfied with the assistance provided by the palliative care team regarding goals of care elucidation."

Provider Attitudes Survey
COMIRB # 24-1913
PI: Sydney Ariagno, MD
Version: 1.0
Version Date: 10-29-2024

"I am satisfied with the assistance provided by the palliative care team regarding advance care planning."
"I am satisfied with the assistance provided by the palliative care team regarding high-stakes medical decision making."
"I am satisfied with the assistance provided by the palliative care team regarding care conference management."
"I am satisfied with the assistance provided by the palliative care team regarding code status discussions."
"I am satisfied with the assistance provided by the palliative care team regarding end-of-life planning."

<u>Attitudes Towards Timing of Palliative Care Involvement</u>
Please select the option that best describes the ideal time that palliative care should first be consulted for a patient undergoing allogeneic bone marrow transplant:

- At the time that BMT is being discussed as a possible treatment option
- At the time of initial BMT consultation
- At the time of admission for BMT
- If pain or symptom management during BMT is a problem
- If a patient develops a serious problem related to BMT or if their underlying disease returns after BMT
- If a patient requires intensive care unit admission following BMT
- If a patient is dying following BMT
- The palliative care team should never be involved in a child's BMT care

<u>Provider Perceptions Regarding How Families Receive Palliative Care Referrals</u>
Please indicate the degree to which you agree with the following statements (response options include: strongly disagree, disagree, somewhat disagree, neither agree nor disagree, somewhat agree, agree, and strongly agree)

When families hear the term "palliative care", they feel:
Scared
Hopeful
Stressed
Secure
Depressed
Anxious
Reassured

Please indicate the degree to which you agree with the following statements (response options include: strongly disagree, disagree, somewhat disagree, neither agree nor disagree, somewhat agree, agree, and strongly agree)

If I recommend a palliative care referral for my patient and their family, they might…
Think the more support they get, the better they would feel
Think their child's disease was out of control

Provider Attitudes Survey
COMIRB # 24-1913
PI: Sydney Ariagno, MD
Version: 1.0
Version Date: 10-29-2024

Think I really care about what is happening to us
Think nothing more can be done for their child's disease
Think I have given up on them
Think their child's disease is terminal
Think more positively about the future
Feel more in control of their situation
Worry the palliative care team would talk to them about dying
Worry the palliative care team would interfere with their child's transplant therapy
Worry the palliative care team would interfere with their relationship with the BMT team
Feel less hopeful for a successful transplant
