# Supplementary material for: Perceptions of Proactive Palliative Care Integration Among Pediatric Hematopoietic Cell Transplant Providers: A Pilot Study
Source: Children (Basel). 2026 Jun 26;13(7):854. doi: 10.3390/children13070854 (PMC13406733; doi:10.3390/children13070854)
Supplement: Supplementary file 1 [file children-13-00854-s001.zip › children-4360387-File S2.pdf]

Supplemental File S2 – Raw Survey Data

AIM and IAM Survey Results

The responses in this section are scored on a 5-point Likert scale with 1 representing “completely disagree” and 5 representing “strongly agree”.

**Please indicate the degree to which you agree with the following statements:**	**N = 18**[1]
**Standardized palliative care integration for allogeneic BMT patients meets my approval**	
Completely disagree	0 (0%)
Disagree	0 (0%)
Neither agree nor disagree	0 (0%)
Agree	2 (11%)
Strongly agree	16 (89%)
**Standardized palliative care integration for allogeneic BMT patients is appealing to me**	
Completely disagree	0 (0%)
Disagree	0 (0%)
Neither agree nor disagree	0 (0%)

Please indicate the degree to which you agree with the following statements:	N = 18[1]
Agree	1 (5.6%)
Strongly agree	17 (94%)
**I like standardized palliative care integration for allogeneic BMT patients**	
Completely disagree	0 (0%)
Disagree	0 (0%)
Neither agree nor disagree	0 (0%)
Agree	0 (0%)
Strongly agree	18 (100%)
**I welcome standardized palliative care integration for allogeneic BMT patients**	
Completely disagree	0 (0%)
Disagree	0 (0%)
Neither agree nor disagree	0 (0%)
Agree	0 (0%)

**Please indicate the degree to which you agree with the following statements:**	**N = 18**[1]
Strongly agree	18 (100%)
**Standardized palliative care integration for allogeneic BMT patients seems fitting**	
Completely disagree	0 (0%)
Disagree	0 (0%)
Neither agree nor disagree	0 (0%)
Agree	1 (5.6%)
Strongly agree	17 (94%)
**Standardized palliative care integration for allogeneic BMT patients seems suitable**	
Completely disagree	0 (0%)
Disagree	0 (0%)
Neither agree nor disagree	0 (0%)
Agree	1 (5.6%)
Strongly agree	17 (94%)

Please indicate the degree to which you agree with the following statements:	N = 18[1]
**Standardized palliative care integration for allogeneic BMT patients seems applicable**	
Completely disagree	0 (0%)
Disagree	0 (0%)
Neither agree nor disagree	0 (0%)
Agree	1 (5.6%)
Strongly agree	17 (94%)
**Standardized palliative care integration for allogeneic BMT patients seems like a good match**	
Completely disagree	0 (0%)
Disagree	0 (0%)
Neither agree nor disagree	0 (0%)
Agree	2 (11%)
Strongly agree	16 (89%)

[1] n (%)

Provider satisfaction with recommendations from SPC

The response categories are ordered from most strong agreement to most strong disagreement out of the responses received. If a category is missing, no providers chose that option for that question. These questions are scored on a 7-point Likert scale with 1 representing “strongly disagree” and 7 representing “strongly agree”.

**Please indicate the degree to which you agree with the following statements:**	**N = 18**[1]
**I am satisfied with the recommendations provided by the palliative care team regarding physical symptom management**	
Strongly disagree	0 (0%)
Disagree	0 (0%)
Somewhat disagree	2 (11%)
Neither agree nor disagree	2 (11%)
Somewhat agree	1 (5.6%)
Agree	8 (44%)
Strongly agree	5 (28%)
**I am satisfied with the recommendations provided by the palliative care team regarding emotional symptom management**	
Strongly disagree	0 (0%)

Please indicate the degree to which you agree with the following statements:	N = 18[1]
Disagree	0 (0%)
Somewhat disagree	0 (0%)
Neither agree nor disagree	1 (5.6%)
Somewhat agree	1 (5.6%)
Agree	4 (22%)
Strongly agree	12 (67%)
**I am satisfied with the recommendations provided by the palliative care team regarding communication practices for my patients**	
Strongly disagree	0 (0%)
Disagree	0 (0%)
Somewhat disagree	0 (0%)
Neither agree nor disagree	2 (11%)
Somewhat agree	2 (11%)
Agree	6 (33%)

Please indicate the degree to which you agree with the following statements:	N = 18[1]
Strongly agree	8 (44%)
**I am satisfied with the psychosocial support the palliative care team provides my patients**	
Strongly disagree	0 (0%)
Disagree	0 (0%)
Somewhat disagree	0 (0%)
Neither agree nor disagree	0 (0%)
Somewhat agree	1 (5.6%)
Agree	10 (56%)
Strongly agree	7 (39%)
**I am satisfied with the advocacy provided by the palliative care team regarding patients' needs**	
Strongly disagree	0 (0%)
Disagree	0 (0%)
Somewhat disagree	0 (0%)

Please indicate the degree to which you agree with the following statements:	N = 18[1]
Neither agree nor disagree	0 (0%)
Somewhat agree	2 (11%)
Agree	8 (44%)
Strongly agree	8 (44%)
**I am satisfied with the assistance provided by the palliative care team regarding goals of care elucidation**	
Strongly disagree	0 (0%)
Disagree	0 (0%)
Somewhat disagree	0 (0%)
Neither agree nor disagree	0 (0%)
Somewhat agree	0 (0%)
Agree	4 (22%)
Strongly agree	14 (78%)
**I am satisfied with the assistance provided by the palliative care team regarding advance care planning**	

Please indicate the degree to which you agree with the following statements:	N = 18[1]
Strongly disagree	0 (0%)
Disagree	0 (0%)
Somewhat disagree	1 (5.6%)
Neither agree nor disagree	0 (0%)
Somewhat agree	1 (5.6%)
Agree	3 (17%)
Strongly agree	13 (72%)
**I am satisfied with the assistance provided by the palliative care team regarding high-stakes medical decision making**	
Strongly disagree	0 (0%)
Disagree	0 (0%)
Somewhat disagree	0 (0%)
Neither agree nor disagree	2 (11%)
Somewhat agree	2 (11%)

**Please indicate the degree to which you agree with the following statements:**	**N = 18**[1]
Agree	4 (22%)
Strongly agree	10 (56%)
**I am satisfied with the assistance provided by the palliative care team regarding care conference management**	
Strongly disagree	0 (0%)
Disagree	0 (0%)
Somewhat disagree	1 (5.6%)
Neither agree nor disagree	1 (5.6%)
Somewhat agree	3 (17%)
Agree	4 (22%)
Strongly agree	9 (50%)
**I am satisfied with the assistance provided by the palliative care team regarding code status discussions**	
Strongly disagree	0 (0%)

Please indicate the degree to which you agree with the following statements:	N = 18[1]
Disagree	0 (0%)
Somewhat disagree	1 (5.6%)
Neither agree nor disagree	1 (5.6%)
Somewhat agree	0 (0%)
Agree	4 (22%)
Strongly agree	12 (67%)
**I am satisfied with the assistance provided by the palliative care team regarding end-of-life planning**	
Strongly disagree	0 (0%)
Disagree	0 (0%)
Somewhat disagree	0 (0%)
Neither agree nor disagree	1 (5.6%)
Somewhat agree	0 (0%)

Please indicate the degree to which you agree with the following statements:	N = 18[1]
Agree	3 (17%)
Strongly agree	14 (78%)

[1] n (%)

How providers predict families will feel about SPC

These questions are scored on a 7-point Likert scale with 1 representing “strongly disagree” and 7 representing “strongly agree”.

Please indicate the degree to which you agree with the following statement: When families hear the term ‘palliative care’, they feel…	N = 18[1]
**Scared**	
Strongly disagree	0 (0%)
Disagree	0 (0%)
Somewhat disagree	0 (0%)
Neither agree nor disagree	2 (11%)
Somewhat agree	10 (56%)
Agree	6 (33%)

**Please indicate the degree to which you agree with the following statement: When families hear the term ‘palliative care’, they feel...**	**N = 18**[1]
Strongly agree	0 (0%)
**Hopeful**	
Strongly disagree	1 (5.6%)
Disagree	1 (5.6%)
Somewhat disagree	7 (39%)
Neither agree nor disagree	6 (33%)
Somewhat agree	3 (17%)
Agree	0 (0%)
Strongly agree	0 (0%)
**Stressed**	
Strongly disagree	0 (0%)
Disagree	0 (0%)
Somewhat disagree	1 (5.6%)

**Please indicate the degree to which you agree with the following statement: When families hear the term 'palliative care', they feel...**	**N = 18**[1]
Neither agree nor disagree	4 (22%)
Somewhat agree	7 (39%)
Agree	5 (28%)
Strongly agree	1 (5.6%)
**Secure**	
Strongly disagree	0 (0%)
Disagree	2 (11%)
Somewhat disagree	4 (22%)
Neither agree nor disagree	8 (44%)
Somewhat agree	2 (11%)
Agree	2 (11%)
Strongly agree	0 (0%)
**Depressed**	

**Please indicate the degree to which you agree with the following statement: When families hear the term 'palliative care', they feel...**	**N = 18**[1]
Strongly disagree	1 (5.6%)
Disagree	1 (5.6%)
Somewhat disagree	1 (5.6%)
Neither agree nor disagree	11 (61%)
Somewhat agree	2 (11%)
Agree	2 (11%)
Strongly agree	0 (0%)
**Anxious**	
Strongly disagree	0 (0%)
Disagree	0 (0%)
Somewhat disagree	0 (0%)
Neither agree nor disagree	1 (5.6%)

Please indicate the degree to which you agree with the following statement: When families hear the term 'palliative care', they feel…	N = 18[1]
Somewhat agree	10 (56%)
Agree	6 (33%)
Strongly agree	1 (5.6%)
**Reassured**	
Strongly disagree	0 (0%)
Disagree	2 (11%)
Somewhat disagree	5 (28%)
Neither agree nor disagree	7 (39%)
Somewhat agree	4 (22%)
Agree	0 (0%)
Strongly agree	0 (0%)
**Think the more support they get, the better they would feel**	
Strongly disagree	0 (0%)

Please indicate the degree to which you agree with the following statement: When families hear the term 'palliative care', they feel…	N = 18[1]
Disagree	0 (0%)
Somewhat disagree	0 (0%)
Neither agree nor disagree	0 (0%)
Somewhat agree	7 (39%)
Agree	8 (44%)
Strongly agree	3 (17%)
**Think their child's disease was out of control**	
Strongly disagree	0 (0%)
Disagree	1 (5.6%)
Somewhat disagree	3 (17%)
Neither agree nor disagree	4 (22%)
Somewhat agree	9 (50%)
Agree	1 (5.6%)

Please indicate the degree to which you agree with the following statement: When families hear the term 'palliative care', they feel...	N = 18[1]
Strongly agree	0 (0%)
**Think I really care about what is happening to them**	
Strongly disagree	0 (0%)
Disagree	0 (0%)
Somewhat disagree	2 (11%)
Neither agree nor disagree	2 (11%)
Somewhat agree	6 (33%)
Agree	6 (33%)
Strongly agree	2 (11%)
**Think nothing more can be done for their child's disease**	
Strongly disagree	2 (11%)
Disagree	2 (11%)
Somewhat disagree	4 (22%)
Neither agree nor disagree	4 (22%)

**Please indicate the degree to which you agree with the following statement: When families hear the term 'palliative care', they feel...**	**N = 18**[1]
Somewhat agree	4 (22%)
Agree	2 (11%)
Strongly agree	0 (0%)
**Think their child's disease is terminal**	
Strongly disagree	3 (17%)
Disagree	1 (5.6%)
Somewhat disagree	3 (17%)
Neither agree nor disagree	4 (22%)
Somewhat agree	5 (28%)
Agree	2 (11%)
Strongly agree	0 (0%)
**Think more positively about the future**	
Strongly disagree	0 (0%)
Disagree	3 (17%)

Please indicate the degree to which you agree with the following statement: When families hear the term 'palliative care', they feel...	N = 18[1]
Somewhat disagree	4 (22%)
Neither agree nor disagree	7 (39%)
Somewhat agree	1 (5.6%)
Agree	3 (17%)
Strongly agree	0 (0%)
**Feel more in control of their situation**	
Strongly disagree	0 (0%)
Disagree	1 (5.6%)
Somewhat disagree	2 (11%)
Neither agree nor disagree	6 (33%)
Somewhat agree	5 (28%)
Agree	4 (22%)
Strongly agree	0 (0%)

Please indicate the degree to which you agree with the following statement: When families hear the term ‘palliative care’, they feel…	N = 18[1]
**Worry the palliative care team would talk to them about dying**	
Strongly disagree	1 (5.6%)
Disagree	0 (0%)
Somewhat disagree	0 (0%)
Neither agree nor disagree	6 (33%)
Somewhat agree	8 (44%)
Agree	3 (17%)
Strongly agree	0 (0%)
**Worry the palliative care team would interfere with their child's transplant therapy**	
Strongly disagree	7 (39%)
Disagree	4 (22%)
Somewhat disagree	5 (28%)
Neither agree nor disagree	2 (11%)

Please indicate the degree to which you agree with the following statement: When families hear the term ‘palliative care’, they feel…	N = 18[1]
Somewhat agree	0 (0%)
Agree	0 (0%)
Strongly agree	0 (0%)
**Worry the palliative care team would interfere with their relationship with the BMT team**	
Strongly disagree	6 (33%)
Disagree	4 (22%)
Somewhat disagree	5 (28%)
Neither agree nor disagree	3 (17%)
Somewhat agree	0 (0%)
Agree	0 (0%)
Strongly agree	0 (0%)
**Feel less hopeful for a successful transplant**	
Strongly disagree	3 (17%)
Disagree	2 (11%)

**Please indicate the degree to which you agree with the following statement: When families hear the term 'palliative care', they feel...**	**N = 18**[1]
Somewhat disagree	4 (22%)
Neither agree nor disagree	4 (22%)
Somewhat agree	4 (22%)
Agree	1 (5.6%)
Strongly agree	0 (0%)

[1] n (%)
